# Improvement of biohistological response of facial implant materials by tantalum surface treatment

**DOI:** 10.1186/s40902-019-0231-3

**Published:** 2019-11-27

**Authors:** Mohammed Mousa Bakri, Sung Ho Lee, Jong Ho Lee

**Affiliations:** 1 Oral and Maxillofacial Surgery Department, Seoul National University Dental Hospital, Seoul, South Korea; 2 Oral and Maxillofacial Surgery Department, School of Dentistry, Jazan University , Jazan City, Saudi Arabia; 3 Department of Oral and Maxillofacial Surgery, School of Dentistry, Seoul National University Dental Hospital, Daehakro 101, Jongro-Gu, Seoul, 03080 South Korea; 4 Department of Oral and Maxillofacial Surgery, Dental Research Institute, School of Dentistry, Seoul National University, Seoul, South Korea; 5 Department of Oral and Maxillofacial Surgery, Seoul National University Dental Hospital, 275-1 Yeongeon-dong, Jongno-gu, Seoul, 110-749 South Korea

**Keywords:** Tantalum ion implantation, Surface treatment, ePTFE, Silicone

## Abstract

**Background:**

A compact passive oxide layer can grow on tantalum (Ta). It has been reported that this oxide layer can facilitate bone ingrowth in vivo though the development of bone-like apatite, which promotes hard and soft tissue adhesion. Thus, Ta surface treatment on facial implant materials may improve the tissue response, which could result in less fibrotic encapsulation and make the implant more stable on the bone surface. The purposes of this study were to verify whether surface treatment of facial implant materials using Ta can improve the biohistobiological response and to determine the possibility of potential clinical applications.

**Methods:**

Two different and commonly used implant materials, silicone and expanded polytetrafluoroethylene (ePTFE), were treated via Ta ion implantation using a Ta sputtering gun. Ta-treated samples were compared with untreated samples using in vitro and in vivo evaluations. Osteoblast (MG-63) and fibroblast (NIH3T3) cell viability with the Ta-treated implant material was assessed, and the tissue response was observed by placing the implants over the rat calvarium (*n* = 48) for two different lengths of time. Foreign body and inflammatory reactions were observed, and soft tissue thickness between the calvarium and the implant as well as the bone response was measured.

**Results:**

The treatment of facial implant materials using Ta showed a tendency toward increased fibroblast and osteoblast viability, although this result was not statistically significant. During the in vivo study, both Ta-treated and untreated implants showed similar foreign body reactions. However, the Ta-treated implant materials (silicone and ePTFE) showed a tendency toward better histological features: lower soft tissue thickness between the implant and the underlying calvarium as well as an increase in new bone activity.

**Conclusion:**

Ta surface treatment using ion implantation on silicone and ePTFE facial implant materials showed the possibility of reducing soft tissue intervention between the calvarium and the implant to make the implant more stable on the bone surface. Although no statistically significant improvement was observed, Ta treatment revealed a tendency toward an improved biohistological response of silicone and ePTFE facial implants. Conclusively, tantalum treatment is beneficial and has the potential for clinical applications.

## Background

Facial augmentation procedures are some of the most commonly performed cosmetic procedures [1]. Silicone rubber and silicone rubber-based materials are widely used in facial cosmetics. This has been the case for many years. However, there is increasing evidence suggesting that the intrinsically hydrophobic nature of the silicone rubber surface leads to poor cell adhesion and tissue compatibility between the implant and surrounding tissues, which results in capsule formation and gradual thickening and contracture of these tissues [2, 3]. In addition, these capsular voids encourage bacterial infection and invasion as well as inflammation during long-term use.

ePTFE, which is a polymer that was invented by W.L. Gore Company and has been safely used as facial implant material with several clinical applications for more than 10 years, if necessary, is relatively easy to remove [4]. However, an unsatisfactory appearance may result from the implant folding on itself due to the mobility of the underlying tissues. Therefore, the major disadvantages of using facial implants continue to be susceptibility to infection and possible displacement. Thus, there is a need for further research into ways to improve the outcome of facial implant use. Surface modification of implant materials is a commonly used method to improve the biocompatibility of implant materials and to overcome the abovementioned disadvantages. Various materials have been used as implant coatings.

Tantalum has been receiving increasing interest as a biomaterial due to its excellent biocompatibility (e.g., outstanding bone-like apatite forming capability, absence of cytotoxic ion release or dissolution in local, systemic, and remote organs, and good osseointegration), superior strength, and anti-corrosion properties [5]. Tantalum is a corrosion-resistant transition metal element with atomic number 73. It is a promising metallic material, at least in terms of bioperformance. It has been used in the medical field since the 1940s when Burke used pure tantalum in several cases, such as in the skin, in the subcutaneous and tendon sutures, and in several plates [6]. Tantalum has the ability to form a compact, passive, extremely thin, and transparent but strong and tenacious oxide layer that strongly adheres to tantalum. This oxide layer has the capacity to facilitate bone ingrowth under in vivo conditions via the development of bone-like apatite that promotes hard and soft tissue adhesion [7]. Furthermore, Ta is a hard, ductile, and highly chemical-resistant material with good apposition to human bone. In addition, the mechanical properties of tantalum are impressive. The metal is comparable to steel in its strength, toughness, and workability [8, 9]. The aim of this study was (1) to verify whether the use of tantalum as a surface treatment material for facial implant materials can improve the biohistobiological features and (2) to determine the possibility for potential clinical applications.

## Materials and methods

### Tantalum ion implantation

A 0.85-mm-thick ePTFE membrane (Meari Co., Ltd., Gyeonggi-do, Republic of Korea) and a 1-mm-thick silicone rubber consisting of clinical-grade silicone (Bistool Co. Ltd., Seoul, Republic of Korea) with dimensions of 10 × 10 mm was prepared for the experimental evaluation. All samples were ultrasonically cleaned in alcohol and deionized water for 5 min before processing with tantalum (Ta) coating. A Ta target (diameter 75 mm, thickness 5 mm, purity 99.99%, Kojundo Korea Co., Ltd., Gyeonggi-do, Republic of Korea) was placed in a DC magnetron sputter gun housing (Ultech Co. Ltd., Daegu, Korea). The vacuum chamber was pumped to 5 × 10^−4^ Pa using rotary and diffusion pumps. To generate a sufficient amount of Ta ions and neutral atoms, 25 W of target power was applied to the Ta sputtering gun, and a working pressure and temperature were maintained at 0.6 Pa and 25 °C, respectively, during the process. The samples were placed on a stainless steel plate parallel to the Ta target surface at a 100-mm distance. Ta ions and neutral atoms were implanted into the sample surfaces for 3 min using a high negative bias of 2000 V. For comparative purposes, only untreated ePTFE and untreated silicone rubber sheets were used for the control group. Thus, all of the samples in this study were divided into the following four groups: Ta-treated silicone implant (G1), untreated silicone implant (G2), Ta-treated ePTFE implant (G3), and untreated ePTFE implant (G4).

### TEM and SEM observation

To observe the Ta-implanted regions on the implant surfaces, high-resolution transmission electron microscope images were collected using transmission electron microscopy (TEM) (JEM-2100F, JEOL, Japan) operated at 200 kV. The cross-sectional image of the Ta-coated implant surface was prepared and obtained using focused ion beam milling and field-emission scanning electron microscopy (FIB/FE-SEM) (AURIGA, Carl Zeiss, Germany). Prior to the milling process, protective layers containing platinum and carbon were coated onto the implant surfaces.

### Cell viability

To evaluate the viability of osteoblasts (MG-63) and fibroblasts (NIH3T3), an EZ-Cytox assay (Daeil Lab Service Co. Std., Seoul, Korea) was performed according to the manufacturer’s protocol. MG-63 and NIH3T3 cells were plated at a density of 3 × 10^4^ cells/mL on the implant materials and cultured in Dulbecco’s modified Eagle’s medium (DMEM, ATCC 30-2003) (Life Technologies Co., Grand Island, NY, USA) and Eagle’s minimum essential medium (EMEM, Gibco 11995) (Life Technologies Co.) with 10% fetal bovine serum (FBS) containing 1% penicillin/streptomycin at 37 °C under 5% CO_2_ in a humidified atmosphere. Implants were conditioned in a medium for 5 h before insertion into 24-well cell culture plates. After culturing for 24, 48, or 72 h, the culture medium was discarded, and the samples were washed three times with phosphate-buffered saline (PBS) and then incubated at 37 °C for another 4 h in fresh culture medium containing 10 μL of EZ-Cytox solution. To investigate the effects of the tantalum-treated or untreated silicone or ePTFE surface on the viability of MG-63 and NIH3T3, the absorbance values of each cell culture were measured by using a spectrophotometer (BioTek Instruments, Inc., Winooski, VT, USA) at 490 nm. Each test was repeated four times (*n* = 6).

### Animal study

For biohistological evaluation, an animal experiment was conducted. All experimental surgical procedures were performed in a specific pathogen-free unit. The animals, 6-week-old male healthy Sprague-Dawley rats, were kept in a room with a 12-h light/dark cycle and temperature that varied between 23 and 25 °C. Furthermore, the animals were housed in soft, sterile bedding that was free from antibacterial products. The animals had open access to food and sterile non-acidic water.

The rats were randomly assigned to one of the following groups:

- G1 (*n* = 6): tantalum-treated silicone implant
- G2 (*n* = 6): untreated silicone implant
- G3 (*n* = 6): tantalum-treated ePTFE implant
- G4 (*n* = 6): untreated ePTFE implant

**Fig. 1:**
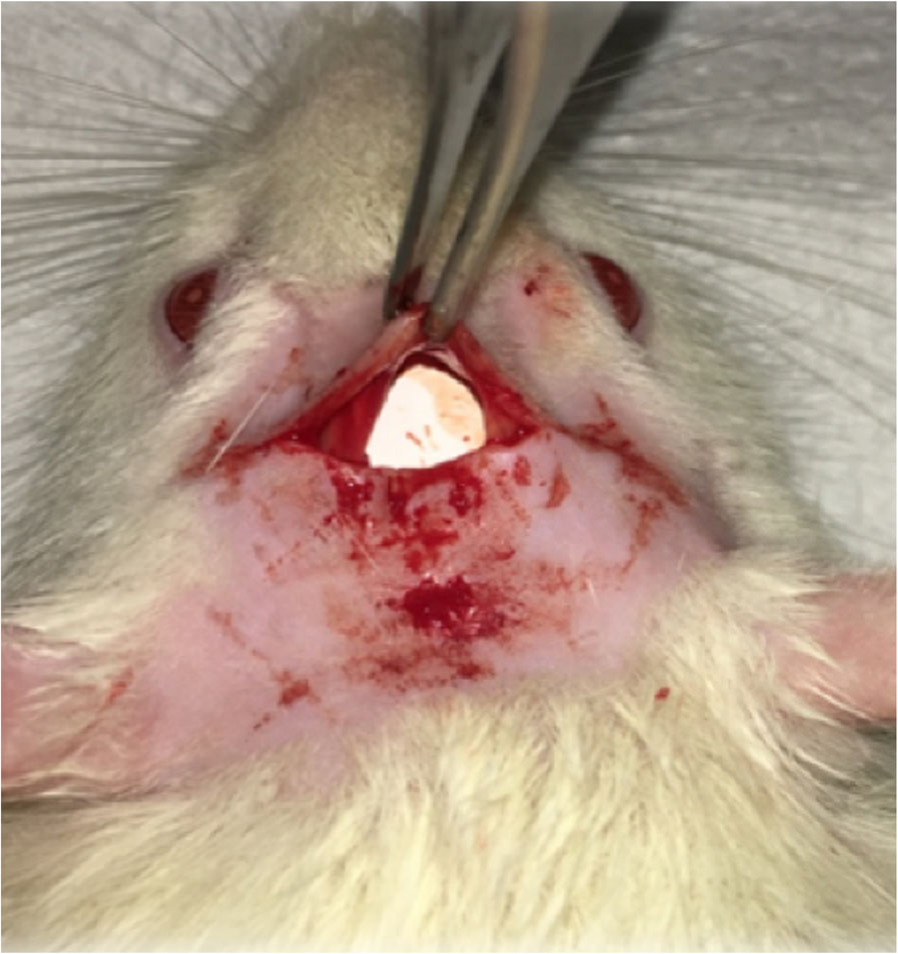
Intraoperative photograph. Intraoperative photograph showing the implant material adapted to the calvarium before suturing. The rat’s head was shaved and disinfected prior to making an incision. A 2-cm-long transverse incision was made on the rat’s calvarium. Then, the implant was placed subperiosteally over the calvaria

The experiment was conducted over two time intervals: 4 and 8 weeks for the silicone implant material and 2 and 4 weeks for the ePTFE implant material. The animals were anesthetized using a ketamine/xylazine mixture (75–100 mg/kg ketamine + 5–10 mg/kg xylazine), which was administered intraperitoneally (IP) using a maximum dose of 10 mL/kg. The incision site, which was located behind the lambdoid suture, was shaved and painted using iodine swabs. An approximately 2-cm-long incision was made behind the lambdoid suture through the skin, subcutaneous tissue, deep fascia, and periosteum up to the calvarial bone. The soft tissue over the skull bone was reflected, and the implants were inserted into all animals in all four groups (Fig. 1). Skin apposition was achieved using subcutaneous sutures made from 4-0 Vicryl (Ethicon, Livingston, UK).

### Histological evaluation

At the completion of the study, the rats were sacrificed using an overdose of an intraperitoneal ketamine/xylazine mixture. Histological samples, including implants and the surrounding tissues, were obtained carefully to prevent implant movement. The samples were fixed in buffered formalin for 24 h, dehydrated, and embedded in paraffin wax. Tissue sections were mounted on glass slides and stained with hematoxylin and eosin (H&E) for histopathological evaluation. Images were captured using a specialized system, SPOT RTTM-KE color mosaic, and digitized via SPOT software version 4.6 (Diagnostic Instruments, Inc., Sterling Heights, MI, USA). For simplicity, images were studied at × 400 magnification. The soft tissue thickness between the implants and the bone, the extent of new bone formation, and the severity of the inflammatory reaction were measured and analyzed.

The sections were processed, placed on slides, and stained with hematoxylin and eosin stain. Histological evaluation was performed on each section to evaluate the inflammation, foreign body reaction, amount of soft tissue filling the gap between the implants and the calvarial bone, and newly formed bone along the superficial layer of the calvarium toward the implants. The inflammation and foreign body reaction were blindly evaluated by a board-certified pathologist. According to the method of Pinese et al., soft tissue measurements were taken at 13 random regions along the implant-bone gap and then averaged to compare Ta-treated and untreated implants [10]. To evaluate the newly formed bone, histological evaluation of the slides was conducted. The newly formed bone was evaluated and scored according to its quantity. The following scores were assigned, as appropriate: no bone (score = 0), little stumps of bone (score = 1), moderate bone with gaps (score = 2), and complete bone along the calvarium surface (score = 3) [11].

### Statistical analysis

All data are expressed as the mean ± standard deviation (SD). Data analysis was conducted using the SPSS Statistics software ver. 25 (IBM, Armonk, NY, USA). Nonparametric data comparisons were performed using the Wilcoxon-Mann-Whitney *U* test. Statistical significance was set at *p* < 0.05.

## Results

### Tantalum ion implantation

**Fig. 2:**
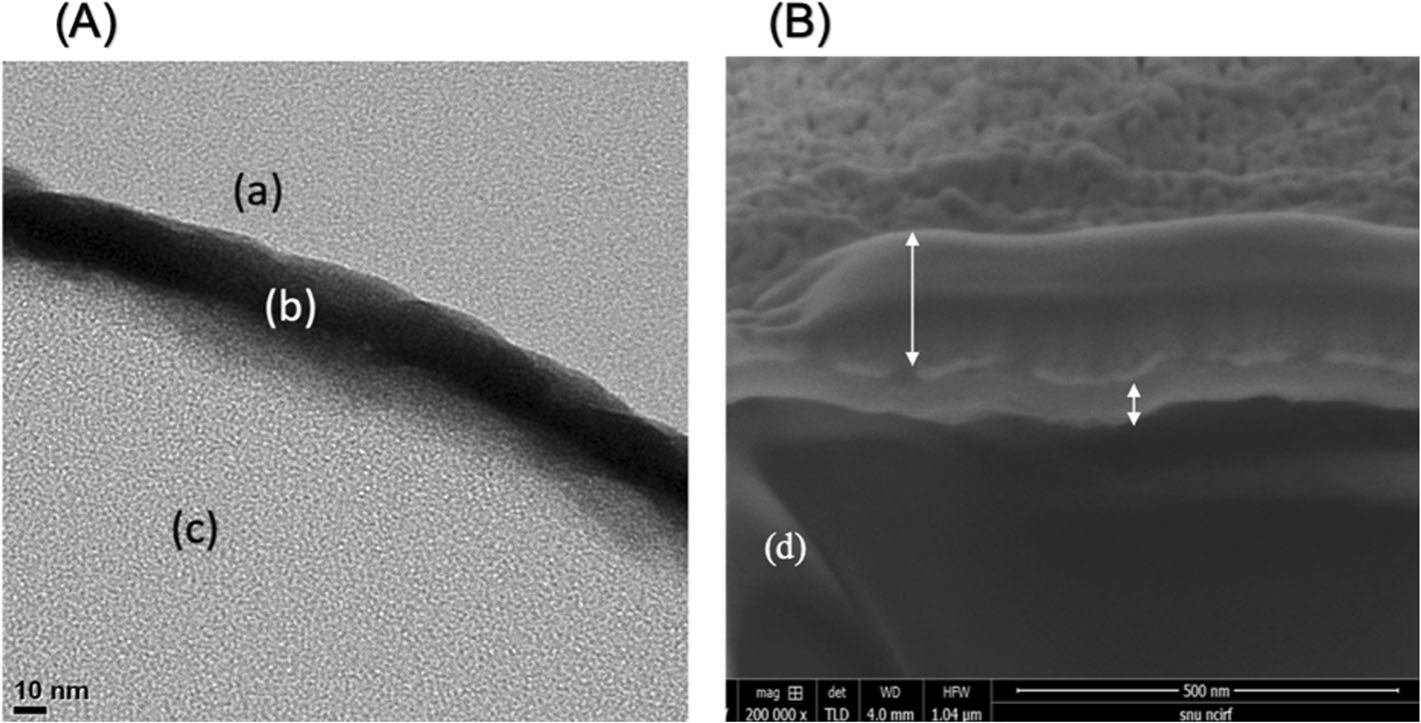
Transmission and scanning electron microscopy images of a tantalum implanted ePTFE implant material. **A** Transmission electron microscopy images: (a) A protective carbonic layer was formed on the implant material surface after treatment with Ta. (b) Ta layer after implantation. (c) ePTFE implant material. **B** Scanning electron microscope images: long two-headed arrows, protective carbonic layer. Short two-headed arrow, Ta layer after ion implantation. (d) ePTFE implant material

After surface modification via Ta ion implantation, the Ta element was observed on the surface of the implant materials. In the TEM cross-sectional images (Fig. 2), a Ta-implanted region with a 20~30-nm thickness was clearly detected between the surfaces of the implants and the protective carbon coating layer.

### Cell viability

The mean absorbance value at a wavelength of 450 nm for the Ta-treated silicone implant materials was 0.060 ± 0.013, and for the untreated silicone implants, the value was 0.054 ± 0.040. The OD value was higher for the Ta-treated silicone implant material; however, the result was not statistically significant (*p* value = 0.4). The ODs for Ta-treated silicone and untreated silicone were 0.089 ± 0.034 and 0.062 ± 0.023, respectively, with a *p* value = 0.4. The result was better than that for untreated silicone, but not statistically significant (Table 1).

**Table 1:** Cell viability assessment

	Mean ± SD	*p* value	Mean ± SD	*p* value
Group	Fibroblast		Osteoblast	
Ta-treated silicone	0.060 ± 0.013	0.4	0.173 ± 0.101	0.7
Untreated silicone	0.054 ± 0.008		0.128 ± 0.070	
Ta-treated ePTFE	0.089 ± 0.034	0.4	0.349 ± 0.285	0.7
Untreated ePTFE	0.062 ± 0.023		0.202 ± 0.081	

The optical density was used to measure the cell viability of fibroblasts and osteoblasts at a wavelength of 450 nm. For Ta-treated silicone implant materials, the OD (0.060 ± 0.013) was higher than that of untreated silicone implant materials (0.054 ± 0.008) (*p* = 0.4). In the case of Ta-treated ePTFE, the tantalum-treated implants showed results (0.089 ± 0.034) that were comparable with the untreated implants (0.062 ± 0.023), *p* value = 0.4. The results were not statistically significant. In the case of osteoblast viability, even though the OD measurements were higher for the Ta-treated implant materials compared with untreated materials, the results were not statistically significant.

### Histological evaluation

**Fig. 3:**
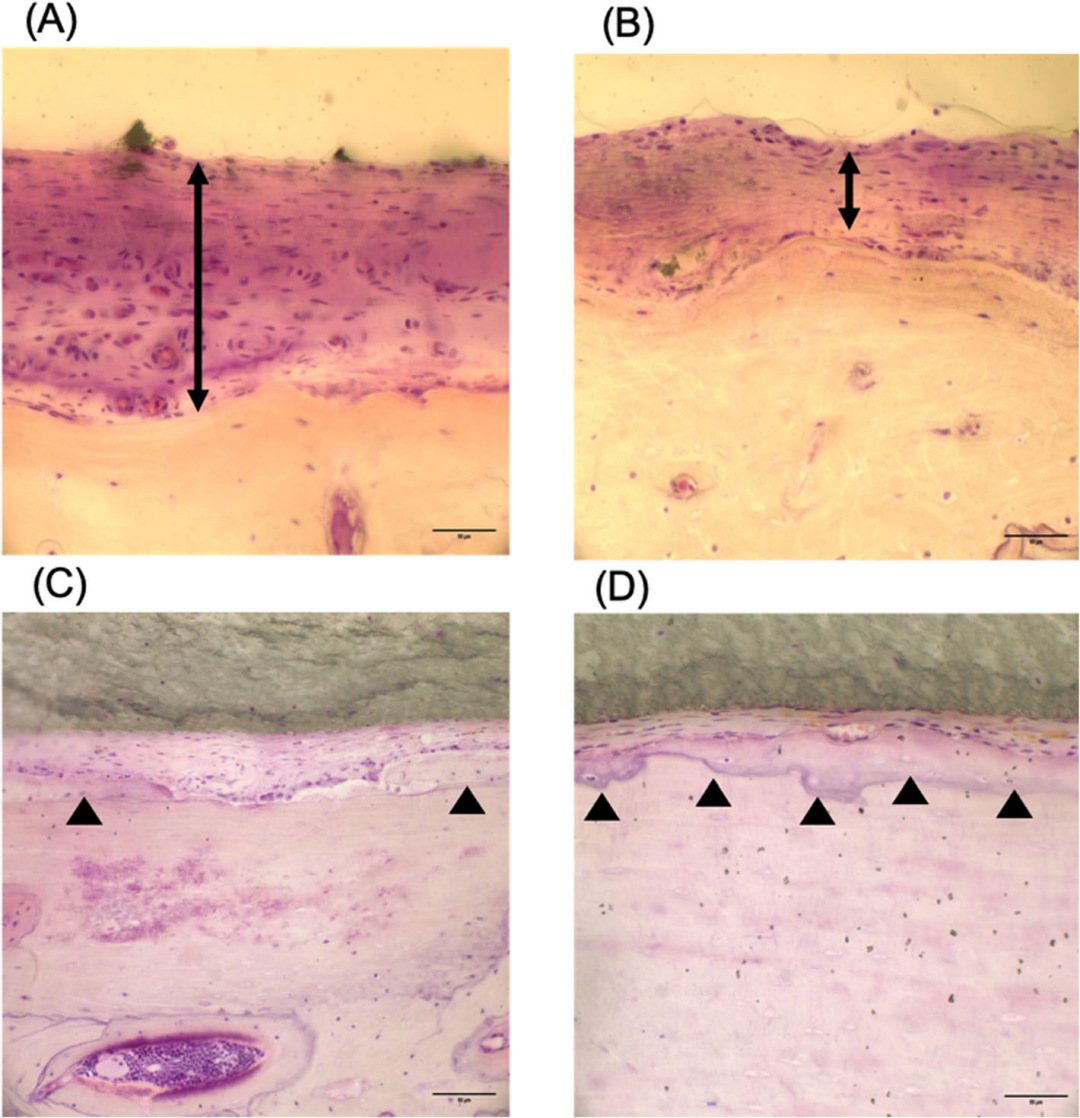
Photomicrographs of the histological slides. Photomicrographs of the histological slides showing biological responses toward Ta-treated and untreated facial implants. **a** Untreated silicone implant material and **b** Ta-treated silicone implant material. The two-headed arrows in **a** and **b** indicate the soft tissue thickness between the implant material and the bone at 8-week intervals. **c** Untreated ePTFE implant and **d** treated ePTFE implant material. The upwards filled arrows in **c** and **d** indicate newly formed bone between the implant material and the bone at 4-week intervals (H&E stain, × 400)

All animals recovered uneventfully after the implantation. There were no cases of death, swelling, or pus discharge at the implant sites in any of the animals during the study period. According to a report by a pathologist, macrophages and gain cells were observed in a short-term experiment. However, in a long-term experiment, none of these cells were observed (Fig. 3). The soft tissue filling the gap between the implant and the calvarium bone was evaluated in all specimens, and the mean ± standard deviation (SD) was recorded. Comparisons were performed using the Wilcoxon-Mann-Whitney *U* test. For short-term Ta-treated silicone implants, the mean soft tissue thickness was 73 ± 34 μm, while the mean thickness for the untreated silicone implant was 92 ± 43 μm. There was less soft tissue filling the gap from the Ta-treated implant material; however, the result was not statistically significant (*p* value = 0.7). In the long-term experiment of silicone implant materials, the Ta-treated implant showed better results compared with untreated implants (Ta-treated 82 ± 27 μm and untreated 115 ± 0.24 μm). However, this result was not statistically significant (*p* value = 0.3). In the case of the newly formed bone in the short-term silicone implant material, we did not observe any newly formed bone from either the Ta-treated silicone implants or the untreated silicone implant materials. In the long-term experiment, a similar amount of newly formed bone was observed in the Ta-treated and untreated silicone implants (0.20 ± 0.45 μm, *p* value = 1.00). Ta coatings did not show a significant improvement compared with the untreated surface (Table 2).

**Table 2:** Histological evaluation of silicone implants

	Soft tissue thickness			New bone formation		
	Mean ± SD		*p* value	Mean ± SD		*p* value
	Ta-treated	Untreated		Ta-treated	Untreated	
4 weeks	73 ± 34	92 ± 43	0.7	0.00	0.00	–
8 weeks	82 ± 27	115 ± 54	0.3	0.20 ± 0.45	0.20 ± 0.45	1.00

Thickness of the soft tissue was expressed as micrometers with the mean and SD reported. In the case of the Ta-treated implant, the thickness was 73 ± 34 μm, while in the untreated group, the thickness was 92 ± 43. The difference was not statistically significant. The *p* value was equal to 0.7. In the long-term study, the *p* value was 0.3, which was not statistically significant. New bone formation in the silicone implant material showed similar results in the short term, with no new bone formation. In the long-term experiment, new bone was observed, and the mean ± SD was almost the same between the Ta-treated and untreated silicone implant material (0.20 ± 0.45).

In the case of the ePTFE implant materials, in the Ta-treated group, the mean soft tissue thickness was 95 ± 71 μm, while the mean thickness for the untreated ePTFE implant was 111 ± 70 μm in the short-term experiment. There was less soft tissue filling the gap in the case of the Ta-treated implant material; however, the result was not statistically significant (*p* value = 0.6). In the long-term experiment of the ePTFE implant materials, the Ta-treated implant did not show any statistically significant differences. For the Ta-treated material, the mean soft tissue thickness was 38 ± 17 μm, and for the untreated implants, the mean soft tissue thickness was 70 ± 63 μm (*p* value = 0.7). In the case of the newly formed bone evaluation, in the short-term experiment, the newly formed bone score was 0.60 ± 0.55 for the Ta-treated implant materials and 0.50 ± 0.58 for the untreated implant materials. The *p* value was 0.80, which means that our result is not statistically significant. In the long-term experiment, the Ta-treated implant material had more newly formed bone (1.40 ± 0.89) compared with the untreated implant material (1.33 ± 1.15). However, the result is not statically significant (*p* value = 0.9) (Table 3).

**Table 3:** Soft tissue thickness in the ePTFE implant materials

	Soft tissue thickness			New bone formation		
	Mean ± SD		*p* value	Mean ± SD		*p* value
	Ta-treated	Non-treated		Ta-treated	Non-treated	
2 weeks	95 ± 71	111 ± 70	0.6	0.60 ± 0.55	0.50 ± 0.58	0.8
4 weeks	38 ± 17	70 ± 63	0.7	1.40 ± 0.89	1.33 ± 1.15	0.9

Soft tissue thickness was expressed as micrometers and reported as the mean ± SD. In the short-term experiment, the thickness in the Ta-treated implant was 95 ± 71 μm, while in the untreated group, the thickness was 111 ± 70 μm. The result was better for the Ta-treated group, but the result was not statistically significant (*p* value = 0.6). In the long-term study, the thickness in the Ta-treated implant was 38 ± 17 μm, while in the untreated group, the thickness was 70 ± 63 μm. Again, the result was better for the Ta-treated group. However, the result was not statistically significant (*p* value = 0.7). New bone formation in the ePTFE implant material showed better results for the Ta-treated implant. However, there was no statistical significance observed in either the short-term (*p* value = 0.6) or long-term (*p* value = 0.6) studies.

## Discussion

Surface treatment of facial implant materials using tantalum (Ta) is considered a promising surface modification technique. Tantalum surface treatment has attracted significant interest because of the ability of tantalum to be used as a surface treatment material via cold spray ion implantation techniques. Cold spray has several advantages over other surface treatment techniques. Specifically, it results in little or no oxidation during material buildup and it produces a dense coating. The process can provide a relatively high deposition efficiency, and the process is conducted in a cold environment, which minimizes any deleterious effects on the treated material. These characteristics explain the current popularity of tantalum surface treatment [12]. Moreover, tantalum itself as a metal has great biological features. These unique characteristics come from its ability to form a self-passivating surface oxide layer. This external coating layer leads to the formation of a bone-like apatite coating in vivo and affords excellent bone and fibrous ingrowth properties, allowing for rapid and substantial bone and soft tissue attachment [13].

Many studies have used surface modification to enhance the biocompatibility of medical devices (drug-eluting stents, artificial organs, biosensors, catheters, scaffolds for tissue engineering, heart valves, facial augmentation materials, etc.). In orthopedics, tantalum surface treatment is considered a promising surface modification technique. It shows a good effect on cell viability and differentiation. In clinical applications, tantalum has been used as a coating material and has been shown to promote bone ingrowth [14]. Several studies have suggested that tantalum surface treatment has the ability to improve the surface mechanical properties and osteogenic activity of orthopedic devices.

However, there are no studies on Ta-treated facial materials [5].

In this study, two commonly used facial implant materials (silicone and ePTFE) were treated using Ta ion implantation. Silicone is frequently used to augment facial defects. Although it is pliable and easily shaped, it still has some disadvantages that lead to facial implant failure, such as implant displacement and the possibility of infection. Gore-Tex is a trade name of expanded polytetrafluoroethylene (ePTFE). It is safe to use long-term with very rare instances of rejection. Furthermore, ePTFE may be used as a guide membrane. However, a disadvantage of this material is its tendency to fold on itself, which leads to infection [15]. Despite the improvements in facial augmentation techniques, failures and complications still occur [16].

In this experiment, using an in vitro study and optical density measurements, the results of fibroblast cell viability for the Ta-treated silicone (0.060 ± 0.013) implant compared with the untreated implant (0.054 ± 0.008) were found to be statistically insignificant (*p* value 0.4). In addition, a statistically insignificant result was observed for the ePTFE implant materials. Regardless of whether the result is or is not statistically significant, it is a promising result because it proves that tantalum is not harmful to cells.

In this study, osteoblast viability assays were conducted as well. Neither the Ta-treated silicone implant material nor the Ta-treated ePTFE implant material was significantly better than the corresponding control groups. However, the mean osteoblast OD value of Ta-treated implant materials (silicone 0.06, ePTFE 0.09) was better than the mean OD values of the corresponding untreated implant material (silicone 0.05, ePTFE 0.06). However, in general, the result of the Ta-treated material (silicone and ePTFE) was better than that of the untreated implant material. According to a previous result, there was no negative effect on cell viability when using tantalum as a coating material. Researchers have determined that the surface topography of an implanted material is important for morphogenesis. In addition, surface topography affects the biological behavior of cultured cells, such as cell viability and differentiation [17].

In the absence of a foreign body, tissue trauma triggers a series of events that comprise wound healing, i.e., inflammation, viability, and remodeling. The presence of a foreign body interferes with the natural biological response and disrupts the healing process. As a result, we observe a foreign body reaction. The most common histological signs of a foreign body reaction are as follows: an increase in macrophages and gain cell formation, an increase in fibroblast activity, and fibrous encapsulation of the foreign body [18]. In our study, during the daily postoperative period, zero rats showed signs or symptoms of an allergic reaction (e.g., weight loss, delayed wound healing, implant loss, dehiscence, or any other signs).

During histological examination, macrophages and giant cells were observed in the short-term experiments. However, these histological findings were absent in the long-term experiments. These features are a normal body reaction after a wound or a cut [19]. During healing and the inflammatory phase, the migration of blood cells (e.g., phagocytic neutrophils and macrophages) to the wound site is a healthy physiological behavior [20]. Moreover, macrophages were not associated with any other clinical finding. This means that these findings were just a normal response to the surgical intervention.

Capsule contracture is considered to be an inevitable complication of using silicone implants [21]. In this study, the soft tissue filling the gap between the implant and the underlying bone was measured. A decreased thickness means less soft tissue deposition and more completability. In addition, the amount of fibrous tissue in a small layer of soft tissue will be less than the amount of fibrous tissue in a thick soft tissue layer. With no statistical significance, the soft tissue thickness in Ta-treated implant materials was lower than that in the control groups (untreated implant materials). Although the result is not biostatistically strong, we can assume that Ta treatment prevents fibrous capsule formation. It is possible that this advantage is due to the implant’s hydrophilic nature, which was acquired after Ta treatment.

In this study, the soft tissue thickness in the 4-week ePTFE implant material study was lower than that in the 2-week implants. This means that not all soft tissue is due to a fibrotic band. Thus, a long-term experimental study is recommended to verify whether we can achieve definitive results regarding the soft tissue thickness change after tantalum treatment. In the case of the silicone implant material, even though the soft tissue thickness of the Ta-treated group (73 ± 34) was better than that of the untreated group (92 ± 43) after 4 weeks of observation, the 8-week observation result was not better than that of the 4-week study.

## Conclusion

Tantalum surface treatment using ion implantation on silicone and ePTFE facial implant materials showed the possibility of reducing soft tissue intervention between the calvarium and the implant, which results in the implant being more stable on the bone surface. Although statistically significant improvement was observed only for fibroblast viability on the Ta-treated implant, tantalum treatment revealed a tendency toward improving the biohistological response of silicone and ePTFE facial implants. Conclusively, tantalum treatment is beneficial and has the potential for clinical applications.

## Data Availability

Raw data that support the findings of this study is available from the corresponding author [Jong Ho Lee] on request.

## Abbreviations

DMEM: Dulbecco’s modified Eagle medium
EMEM: Eagle’s minimum essential medium
ePTFE: Expanded polytetrafluoroethylene (Gore-Tex)
FBS: Fetal bovine serum
FIB/FE-SEM: Field emission scanning electron microscopy
G1, G2, G3, G4: Group 1, group 2, group 3, group 4
H&E: Hematoxylin and eosin
IP: Intraperitoneal
PBS: Phosphate buffered saline
SD: Standard deviation
Ta: Tantalum
TEM: Transmission electron microscope

## References

[1] Osman RB, Swain MV (2015). A critical review of dental implant materials with an emphasis on titanium. Materials (Basel).

[2] Fischer S, Hirche C, Reichenberger MA, Kiefer J, Diehm Y, Mukundan S, Alhefzi M (2015). Silicone implants with smooth surfaces induce thinner but denser fibrotic capsules compared to those with textured surfaces in a rodent model. PLoS One.

[3] Wick G, Backovic A, Rabensteiner E, Plank N, Schwentner C, Sgonc R (2010). The immunology of fibrosis: innate and adaptive responses. Trends Immunol.

[4] Niamtu J (2006). Advanta ePTFE facial implants in cosmetic facial surgery. J Oral Maxillofac Surg.

[5] Huo WT, Zhao LZ, Yu S, Yu ZT, Zhang PX, Zhang YS (2017). Significantly enhanced osteoblast response to nano-grained pure tantalum. Sci Rep.

[6] Romo T, McLaughlin LA, Levine JM, Sclafani AP (2002). Nasal implants: autogenous, semisynthetic, and synthetic. Facial Plast Surg Clin North Am.

[7] Levine B, Della Valle CJ, Jacobs JJ (2006). Applications of porous tantalum in total hip arthroplasty. J Am Acad Orthop Surg.

[8] Li X, Wang L, Yu X, Feng Y, Wang C, Yang K (2013). Tantalum coating on porous Ti6Al4V scaffold using chemical vapor deposition and preliminary biological evaluation. Korean J Couns Psychother.

[9] Stenlund P, Omar O, Brohede U, Norgren S, Norlindh B, Johansson A (2015). Bone response to a novel Ti-Ta-Nb-Zr alloy. Acta Biomater.

[10] Pinese C, Lin J, Milbreta U, Li M, Wang Y, Leong KW (2018). Sustained delivery of siRNA/mesoporous silica nanoparticle complexes from nanofiber scaffolds for long-term gene silencing. Acta Biomater.

[11] Schallenberger MA, Rossmeier K, Lovick HM, Meyer TR, Aberman HM, Juda GA (2014). Comparison of the osteogenic potential of OsteoSelect demineralized bone matrix putty to NovaBone calcium-phosphosilicate synthetic putty in a cranial defect model. J Craniofac Surg.

[12] Levine B, Sporer S, Della Valle CJ, Jacobs JJ, Paprosky W (2007). Porous tantalum in reconstructive surgery of the knee: a review. J Knee Surg.

[13] Levine BR, Sporer S, Poggie RA, Della Valle CJ, Jacobs JJ (2006). Experimental and clinical performance of porous tantalum in orthopedic surgery. Biomaterials.

[14] Wang Q, Qiao Y, Cheng M, Jiang G, He G, Chen Y (2016). Tantalum implanted entangled porous titanium promotes surface osseointegration and bone ingrowth. Sci Rep.

[15] Patel K, Brandstetter K (2016). Solid implants in facial plastic surgery: potential complications and how to prevent them. Facial Plast Surg.

[16] Brügger OE, Bornstein MM, Kuchler U, Janner SF, Chappuis V, Buser D (2015). Implant therapy in a surgical specialty clinic: an analysis of patients, indications, surgical procedures, risk factors, and early failures. Int J Oral Maxillofac Implants.

[17] Filová E, Bullett NA, Bacáková L, Grausová L, Haycock JW, Hlucilová J (2009). Regionally-selective cell colonization of micropatterned surfaces prepared by plasma polymerization of acrylic acid and 1,7-octadiene. Physiol Res.

[18] Kastellorizios M, Tipnis N, Burgess DJ (2015). Foreign body reaction to subcutaneous implants. Adv Exp Med Biol.

[19] Joe B, Vijaykumar M, Lokesh BR (2004). Biological properties of curcumin-cellular and molecular mechanisms of action. Crit Rev Food Sci Nutr.

[20] Koh TJ, DiPietro LA (2011). Inflammation and wound healing: the role of the macrophage. Expert Rev Mol Med.

[21] Kjøller K, Hölmich LR, Jacobsen PH, Friis S, Fryzek J, McLaughlin JK (2001). Capsular contracture after cosmetic breast implant surgery in Denmark. Ann Plast Surg.

